# Experimental and numerical investigation on micro-motion characteristics of lightweight spacecraft

**DOI:** 10.1371/journal.pone.0353236

**Published:** 2026-07-17

**Authors:** Dongjian Su, Yingjie Yang, Xiaomei Guo, Liqiu Shi, Taoyuan Zhang, Wei Zhang, Jiageng Zheng, Chunlei Jiang

**Affiliations:** 1 School of Mechanical Engineering, Zhejiang University of Water Resources and Electric Power, Hangzhou, Zhejiang, China; 2 Engineering University of the Joint Logistics Support Force, PLA, Tianjin, China; 3 School of Mechanical Engineering, Zhejiang University of Water Resources and Electric Power, Hangzhou, Zhejiang, China; 4 School of Mechanical Engineering, Zhejiang University of Water Resources and Electric Power, Hangzhou, Zhejiang, China; 5 School of Mechanical Engineering, Zhejiang University of Water Resources and Electric Power, Hangzhou, Zhejiang, China; 6 School of Mechanical Engineering, Zhejiang University of Water Resources and Electric Power, Hangzhou, Zhejiang, China; 7 School of Mechanical Engineering, Zhejiang University of Water Resources and Electric Power, Hangzhou, Zhejiang, China; 8 School of Energy and Power Engineering, Nanjing University of Science and Technology, Nanjing, Jiangsu, China; Swiss Federal Technology Institute of Lausanne, SWITZERLAND

## Abstract

Accurate prediction of micro-motion characteristics is essential to the success of space mission operations. In this paper, the lightweight spacecraft is selected as the research object. Firstly, the micro-motion characteristic test device is designed to carry out the micro-motion experiment, and the micro-motion characteristic of the test device is numerically simulated to verify the accuracy of the model compared with test data. On this basis, the angular velocity interferences of different values and different directions are simulated, and it is verified that the transversal initial interference is much larger than the spinning angular velocity interference. The cloud images of transversal angular velocity and precession angle under different transversal initial interferences are calculated. The conclusions have some guiding significance for the influence of initial disturbance on the micro-motion characteristics of lightweight spacecraft.

## Introduction

With the rapid advancement of aerospace technology, lightweight spacecraft have demonstrated significant potential in deep space exploration, on-orbit servicing, and distributed satellite systems. These spacecraft exhibit low mass and small moments of inertia, granting them maneuverability but also making them susceptible to disturbances. Initial angular velocity deviations, inevitably introduced during launch separation or actuator operation, can significantly affect their dynamics and induce complex micro-motion characteristics [1–5].

Accurate understanding of micro-motion is crucial for mission success. It directly impacts payload pointing accuracy and serves as a key information source for space object identification. Therefore, investigating the relationship between initial angular velocity deviations and micro-motion characteristics is essential for improving attitude control systems and understanding spacecraft behavior in complex space environments.

This has been studied by several researchers. V. C. Chen from the United States Naval Research Office referred to the vibration, rotation and other motions of the target or its components other than the translational movement of the center of mass as micro-motion [6–8]. F. L. Chernousko gave the expression of the motion of axisymmetric rigid body under the body coordinate system only under the action of gravity [9]. On this basis, W. L. Wang proved that the micro-motion of axisymmetric rigid body only affected by universal gravitation is precession, and studied the change characteristics of attitude angle, spin period, precession angle, precession angle rate and other parameters under the micro-motion of lightweight spacecraft [10]. Junliang Liu used multi-data representation and deep learning algorithm to identify the micro-motion characteristics of ballistic targets [11]. Yizhe Wang established the motion models of spin, precession and nutation of space targets, and used deep convolutional neural networks to classify the forms of micro-motion [12]. R. Z. Zhang proposed a precession parameter estimation of winged warhead based on time-frequency analysis and dual radar, and estimated the precession angle, precession rate and spin rate [13]. J. L. Liu proposed the periodic wave feature to analyze the radiation intensity characteristics of infrared targets and analyze the micro-motion dynamic characteristics of ballistic targets [14].

The initial angular velocity interference generated in the launching process is the main factor affecting the micro-motion characteristics of the lightweight spacecraft. The initial angular velocity interference can be divided into the angular velocity interference in the launching process and the angular velocity interference in the developing process. C. Xu used sensors to record the initial disturbance of the launch system online, and studied the impact of random disturbance on the hit accuracy of the rocket during launch [15]. In order to reduce the initial disturbance of the rocket, Yang Qi optimized the centering part of the rocket, effectively reducing the influence of the initial disturbance [16]. Yuanyuan Li had studied the dynamic characteristics of a plane rigid-flexible coupled solar panel system considering joint gap [17], and ZengBo Liu had studied and built a rigid-flexible coupled dynamic model of spacecraft subjected to multiple disturbances [18]. Liu [19] used an orthogonal experimental technique to study the influence of errors such as the machining gap on the projectile, and Zheng [20] studied the influence of initial state deviation on the flight state of the remote spacecraft.

Pure theoretical analysis faces limitations in solving nonlinear, strongly coupled dynamic equations. Numerical simulation provides an effective approach, but requires rigorous experimental validation. Combining ground testing with high-fidelity simulation has become crucial for systematically analyzing micro-motion responses under different initial angular velocities. This integration represents a key research focus in the field.

Referencing prior research on micro-thrusters by X. Wang [21,22], Z. Zhou [23], and X.G. Jiang [24], a micro-motion test device is designed for a lightweight spacecraft in this study. Experiments are conducted, and a dynamic model for attitude motion is developed. Numerical simulations of the test device’s attitude are performed and compared with experimental results, verifying model accuracy. Simulations under various initial angular velocity deviations reveal their effects on spacecraft attitude. This study aims to deepen understanding of lightweight spacecraft dynamics and provide reliable theoretical and data support for high-precision attitude control design, on-orbit state assessment, and space target characterization.

## Micro-motion characteristic test

### Realization of test device

**Fig 1:**
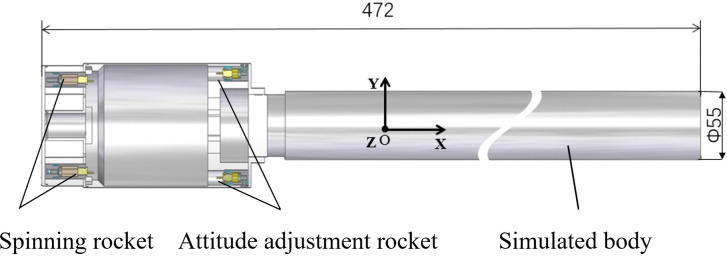
Structure diagram of test device.

As illustrated in Fig 1, the micro-motion characteristic test setup comprises two main components: the micro-motion mechanism and the simulated body. The micro-motion mechanism includes two spinning rockets, two attitude adjustment rockets, and an ignition control module with timing capability. The simulated body is cylindrical in shape and fabricated from 6061 aluminum alloy. A coordinate system is established for the test device, as shown in Fig 1, with its origin located at the center of mass. The OX axis aligns with the longitudinal symmetry axis of the simulated body, while the OY axis lies in the longitudinal symmetry plane and is perpendicular to the OX axis. The OZ axis is determined by the right-hand rule. In this body coordinate system, the axes correspond to the principal axes of inertia. The thrust vectors of the two spinning rockets are oriented along the OZ axis in opposite directions, producing a rolling moment about the OX axis. The thrust vectors of the two attitude adjustment rockets are aligned with the OY axis, generating a pitching moment about the OZ axis.

All rockets utilize an enhanced high-burning-rate dual-base tubular propellant designated GATo-3. For spinning rockets, the propellant dimensions are 8.7 mm outer diameter, 4.7 mm inner diameter, and 13 mm in length. For attitude-adjustment rockets, the propellant measures 7 mm in outer diameter, 4.6 mm in inner diameter, and 5 mm in length. The propellant burning rate r is expressed as


$$\begin{array}{c} =a\overset{n}{\underset{c}{p}} \end{array}$$ (1)

where a is the burning rate coefficient, and $p_{c}$ is the average pressure of the combustion chamber, and n is the burning rate index.

### Test system layout

**Fig 2:**
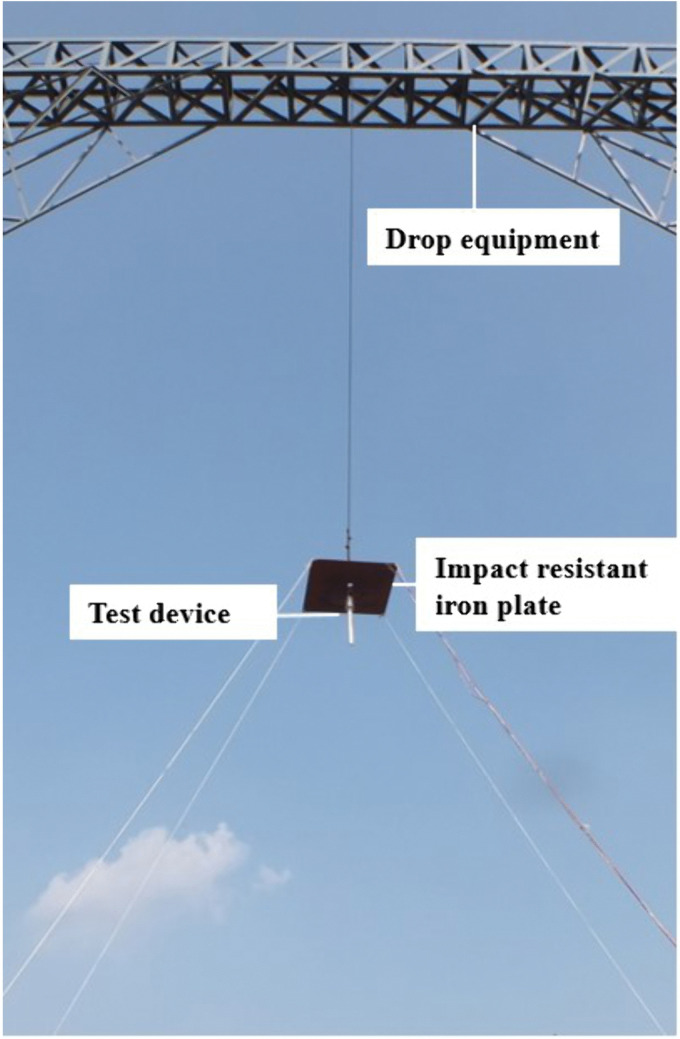
Drop equipment diagram.

Micro-motion testing is conducted outdoors. The micro-motion characteristic test unit is suspended beneath an impact-resistant plate using textile cords; a detailed installation diagram is provided in Fig 2. The drop-test apparatus consists of a drop frame, an impact-resistant steel plate, a detonator, textile cords, and a recovery unit. The detonator ignites a silver cable, which then burns through the textile cords, releasing the test unit into free fall.

**Fig 3:**
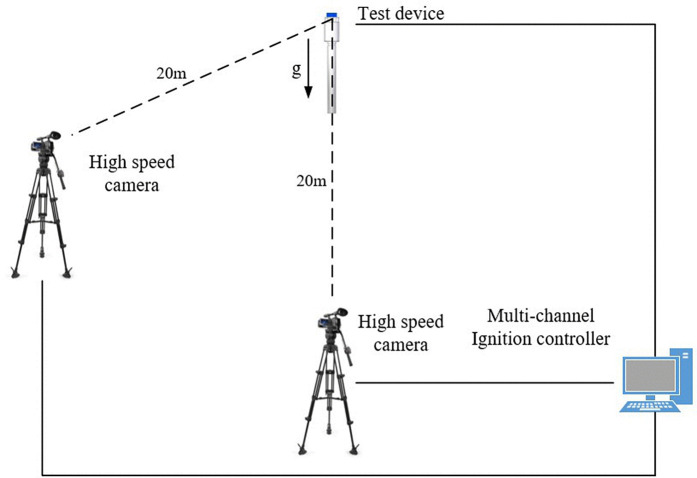
Schematic diagram of the test system.

The layout of the test system is illustrated in Fig 3. Two FastCAM Mini UX50 high-speed cameras are positioned 20 meters away from the simulated projectile, located respectively in front of and to the side of the drop device, to capture the aerial flight posture of the micro-motion test device. These cameras operate at a frame rate of 2000 frames per second with a resolution of 1280 × 1024 pixels. An Xsens MTi-670 inertial measurement unit is mounted on the tail section of the micro-motion test device to measure its attitude during free fall. Upon receiving an external trigger signal, the sensor is activated and begins recording real-time orientation data. A multi-channel ignition controller is employed to provide synchronized electrical signals for the detonators, attitude sensors, and the triggering mechanisms of both high-speed cameras. This controller enables precise timing control across multiple channels, delivering electrical outputs at predetermined intervals to ensure proper test execution and data integrity. This test is a proof-of-concept type.

### Pulsed rocket thrust measurement

**Fig 4:**
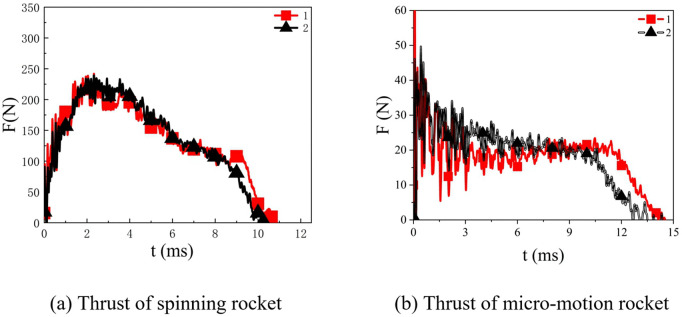
Thrust measurement of pulsed rocket. **(a)** Thrust of spinning rocket. **(b)** Thrust of micro-motion rocket.

Two spinning rockets and two attitude adjusting rockets are all pulsed rockets. The pulsed rocket is an important element for micro-motion test device to change motion attitude during the free fall. In order to reduce the impact of the total pulsed deviation of the rocket, it is necessary to strictly control the loading propellant mass in the pulsed rocket. The attitude adjustment rocket is treated as the micro-motion rocket. The thrust sensors are used to measure the thrust of the two spinning rockets and the two micro-motion rockets respectively, and the results are shown in Fig 4.

In Fig 4(a), the spinning rocket propellant is not coated, and the thrust in reduced surface combustion increased immediately and then decreased continuously until the end of combustion. The mass and total impulse of each propellant are shown in Table 1. As shown in Fig 4(b), the micro-motion rocket propellant is coated, treated as equiplanar combustion. After the ignition propellant ignites the propellant, the thrust rises momentarily and then tends to be flat until the thrust decreases rapidly after the end of combustion, and the mass and total impulse of each charge are shown in Table 2.

**Table 1:** Total impulse and charge mass of two spinning rocket experiments.

No.	Charge mass/g	Total impulse/𝐍·𝐬
**1**	0.8905	1.49276
**2**	0.8853	1.45048

**Table 2:** Total impulse and charge mass of two micro-motion rocket experiments.

No.	Charge mass/g	Total impulse/𝐍·𝐬
**1**	0.1822	0.2569
**2**	0.1803	0.2635

## Micro-motion modelling

The rotation equation about the center of mass in the volume coordinate system is established in Fig 1 as:


$$\begin{array}{c} \dot{H}+\omega \times H=M \end{array}$$ (2)

where ω is the instantaneous rotational angular velocity vector of the lightweight spacecraft, H is the angular momentum vector and can be expressed as H=Iω, I is the inertia matrix of the lightweight spacecraft, and its form is as follows:


$$\begin{array}{c} I\text{=}\left[\begin{array}{ccc} @ccc@I_{x} & -I_{xy} & -I_{xz}\\ -I_{xy} & I_{y} & -I_{yz}\\ -I_{xz} & -I_{yz} & I_{z} \end{array}\right] \end{array}$$ (3)

Then the dynamic equations of the lightweight spacecraft rotating around the principal axis of inertia can be expressed as:


$$\begin{array}{c} \left\{ \begin{array}{c} @l@{I_{x}\dot{\omega }}_{x}-\left({I_{y}-I}_{z}\right)\omega_{y}\omega_{z}=M_{x}\\ {I_{y}\dot{\omega }}_{y}-\left({I_{z}-I}_{x}\right)\omega_{x}\omega_{z}=M_{y}\\ {I_{z}\dot{\omega }}_{z}-(I_{x}-I_{y})\omega_{x}\omega_{y}=M_{z} \end{array}\right. \end{array}$$ (4)

W. L Wang [6] made a detailed study on the dynamic and kinematic characteristics of the lightweight spacecraft that was not acted on by external forces, and obtained that the motion of the lightweight spacecraft could be expressed as follows:


$$\begin{array}{c} \omega \text{=}\left[\begin{array}{c} @l@\omega_{x}\\ \omega_{y}\\ \omega_{z} \end{array}\right]=\left[\begin{array}{c} @l@\omega_{s}\\ \omega_{\text{0}}cos(\Omega_{n}t+\varphi )\\ \omega_{\text{0}}sin(\Omega_{n}t+\varphi ) \end{array}\right] \end{array}$$ (5)

In the body coordinate system, the instantaneous rotational angular velocity vector ω can be expressed as:


$$\begin{array}{c} \begin{array}{c} \omega =\omega_{x}e_{x}+\omega_{y}e_{y}+\omega_{z}e_{z}\mathrm{\backslash hfill}\\ \;\;\;=\omega_{t}+\omega_{x}e_{x}\mathrm{\backslash hfill} \end{array} \end{array}$$ (6)

To further explain the motion of the spin axis of the lightweight spacecraft, the angular momentum vector H of the lightweight spacecraft can be expressed as:


$$\begin{array}{c} \begin{array}{c} H=I_{x}\omega_{x}e_{x}+I_{y}\omega_{y}e_{y}+I_{z}\omega_{z}e_{z}\mathrm{\backslash hfill}\\ \;=I_{t}\omega_{t}+I_{x}\omega_{x}e_{x}\mathrm{\backslash hfill} \end{array} \end{array}$$ (7)

**Fig 5:**
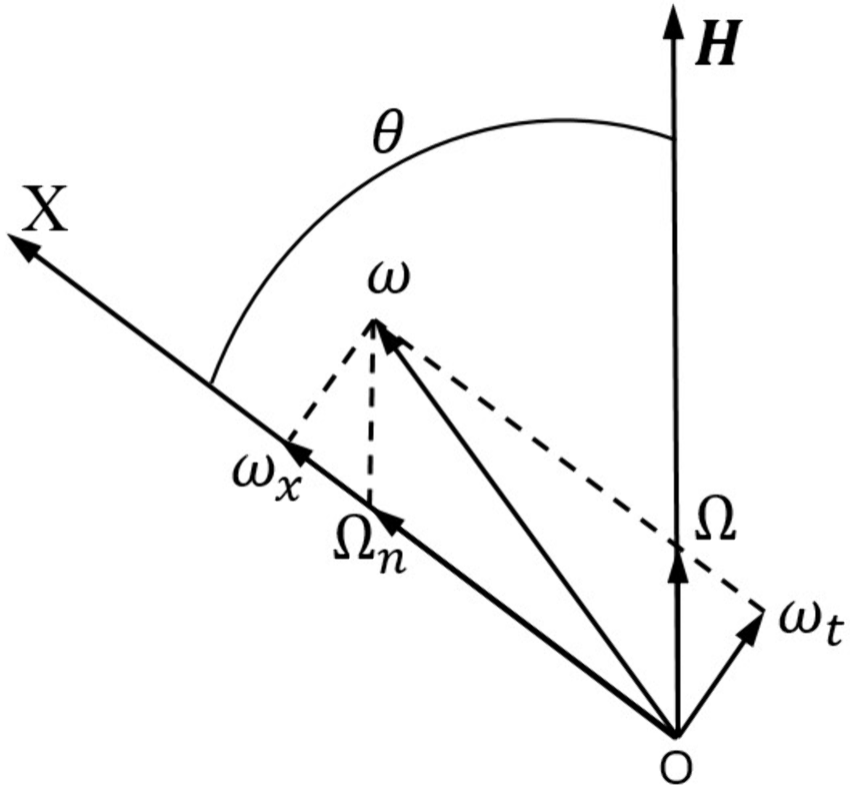
Schematic diagram of angular rate.

Equation (7) shows that the angular momentum vector H consists of two parts: transversal and axial angular velocities. Since the lateral angular velocity $\omega_{t}$ rotates around the spin axis, the spin axis also makes a conical motion, so that the sum vector of the above two parts is oriented in space. This can be obtained by substituting equation (7) into equation (6):


$$\begin{array}{c} \omega =\frac{\text{1}}{I_{t}}H+(1-\frac{I_{x}}{I_{t}})\omega_{x}e_{x}=\frac{H}{I_{t}}e_{k}-\Omega_{n}e_{x} \end{array}$$ (8)

where $e_{k}$ is the unit vector along the direction of angular momentum. From the results of formula (8), it can be seen that the angular velocity vector ω makes two kinds of motion at the same time. One is the conical motion around the OX axis of the device body in the coordinate system, and its rotational speed $\Omega_{n}$ is called the nutation rate of the body. The other one is the conical motion made around the angular momentum vector H, and the rotation speed is $\frac{H}{I_{t}}$, which is called the space nutation rate. Its detailed schematic diagram is shown in Fig 5. The angular momentum vector H, the angular velocity vector ω, and the spin axis OX are three vectors in the same plane.

The angle θ between the OX axis vector and H is called nutation angle. The calculation formula of nutation angle can be expressed as:


$$\begin{array}{c} \theta =arccos\left(\frac{ℋ⬝e_{x}}{H}\right)=arccos\left(\frac{{I_{x}\omega }_{x}}{H}\right) \end{array}$$ (9)

Equation (9) shows that the nutation angle of the axisymmetric spin lightweight spacecraft is constant. Equations (2) ~ (8) describe the equations of lightweight spacecraft motion without external moment, and verify that the axially symmetric lightweight spacecraft is in a stable precession state without external moment. Therefore, it can be concluded that the motion of the form shown in equation (6) is in a stable precession state in a non-static state.

## Analysis of experiment and simulation results

### Experiment results and model verification

**Fig 6:**
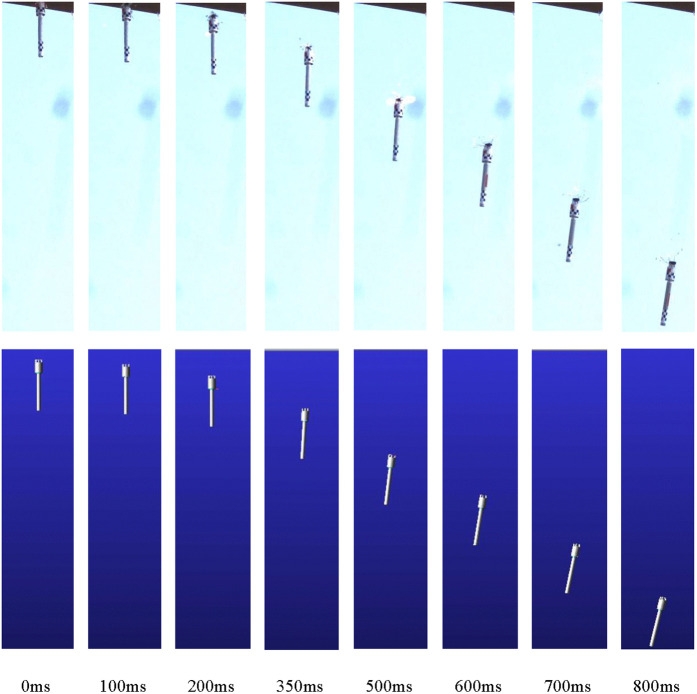
Comparison of experimental and Adams results.

**Fig 7:**
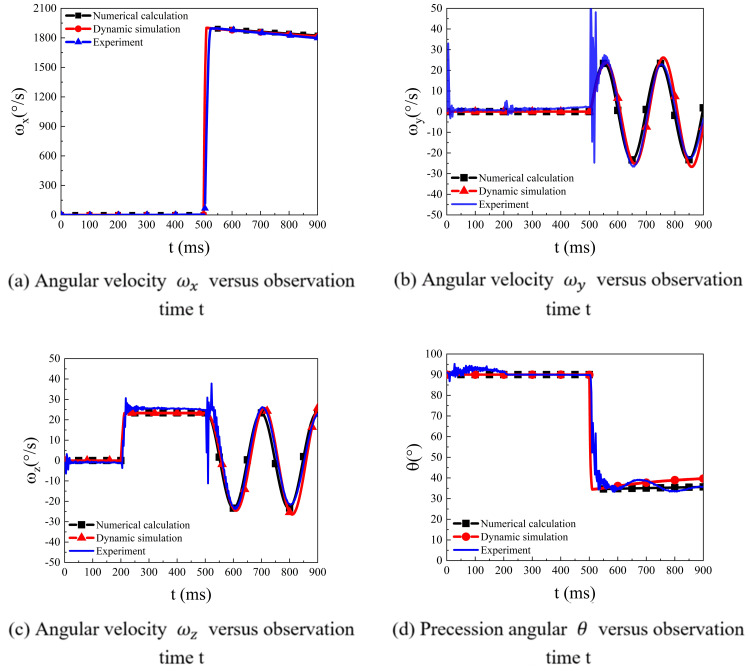
Comparison of simulation and sensor data. **(a)** Angular velocity $\omega_{x}$ versus observation time t. **(b)** Angular velocity $\omega_{y}$ versus observation time t. (c) Angular velocity $\omega_{z}$ versus observation time t. **(d)** Precession angular θ versus observation time t.

Since the test is carried out in the atmosphere and the spinning angular velocity of the micro-motion test device is much higher than the transversal angular velocity, it is necessary to consider the air damping torque in the spin direction of the micro-motion test device and the energy loss caused by the related connection line rotating with the micro-motion test device. Therefore, formula (4) can be written in the following form:


$$\begin{array}{c} \left\{ \begin{array}{c} @l@{I_{x}\dot{\omega }}_{x}-\left({I_{y}-I}_{z}\right)\omega_{y}\omega_{z}=M_{e}\\ {I_{y}\dot{\omega }}_{y}-\left({I_{z}-I}_{x}\right)\omega_{x}\omega_{z}=0\\ {I_{z}\dot{\omega }}_{z}-(I_{x}-I_{y})\omega_{x}\omega_{y}=0 \end{array}\right. \end{array}$$ (10)

where $M_{e}$ is the damping torque subjected to the micro-motion test device. The timing of the ignition controller is set to 200ms when the micro-motion rocket works, simulating the angular velocity interference generated in the launching and development process and set to 500ms spinning rocket work, producing spin stability into the state of micro-motion. The first and second thrust data in Fig 4(a) and the second thrust data in Fig 4(b) are used for the numerical calculation and dynamic simulation to obtain the change of motion attitude of the micro-motion test device at each time point as shown in Fig 6, and the comparison between simulation results and sensor test data is showed in Fig 7.

According to the simulation and test results in Figs 6 and 7, the oscillation of the micro-motion device caused by 0ms detonation would completely disappear within 30ms, but $\omega_{y}$ would produce 1.12°/s angular velocity deviation. At 200ms, the micro pulsed rocket works, and gas is expelled from the nozzle. And the device body rotates around the OZ axis, producing an angular velocity $\omega_{z}$, 25.2°/s. The angular velocity from the numerical calculation and dynamic simulation is 23.22°/s, with an error of 7.94%. At 500ms, the spinning rocket works, and the micro motion test device keeps the stable precession. However, due to the influence of air resistance and energy dissipation, the micro motion test device spin angular velocity continues to decay, resulting in the continuous increase in the change period of the angular velocities $\omega_{y}\text{ and }\omega_{z}$. Because $I_{t}\gg I_{x}$, a small deviation of the lateral angular velocity would lead to a larger deviation of the precession angle, and when $\omega_{t}$ is 25.2°/s, the initial perturbation deviation of the angular velocity would produce a precession angle of 34.9°. Compared with the experimental results, the deviation of precession angle has been maintained in the range of 0% ~ 9.8%, which can verify the accuracy of the model.

### Initial angular velocity perturbation analysis

**Fig 8:**
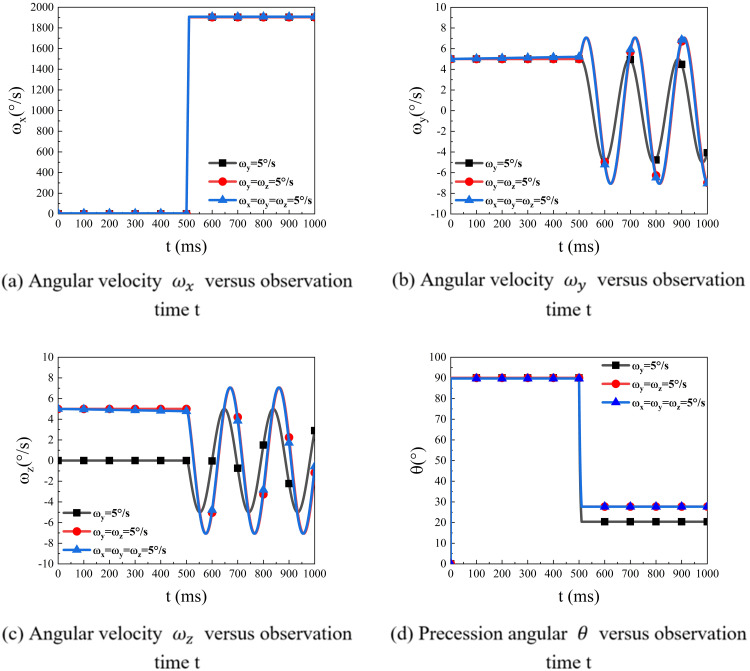
Influence of angular velocity in different directions. **(a)** Angular velocity $\omega_{x}$ versus observation time t. **(b)** Angular velocity $\omega_{y}$ versus observation time t. **(c)** Angular velocity $\omega_{z}$ versus observation time t. **(d)** Precession angular θ versus observation time t.

The experimental results have verified the accuracy of the theoretical model. Because the lightweight spacecraft flies in vacuum environment, the influence of air resistance is ignored, and the whole lightweight spacecraft is regarded as a rigid body, ignoring the influence of energy dissipation. The influence of initial angular velocity deviation in different directions and values on the flying attitude of the lightweight spacecraft is studied respectively. Through experiments, the initial angular velocity of the lightweight spacecraft is not more than 25∘/s. The simulation results obtained by using the thrust of the second spinning rocket in Fig 4(a) through simulation calculation are shown in Fig 8.

As shown in Fig 8(b) and 8(c), the periodic changing curve of $\omega_{z}$ and $\omega_{y}$ with time are presented in the stable precession curve. With the increase of angular velocity $\omega_{z}$, the transversal angular velocity $\omega_{t}$ increases, and the precession angle of the lightweight spacecraft increases significantly. The addition of the angular velocity $\omega_{z}$ in the pitch direction does not affect the periods of the $\omega_{y}\text{ and }\omega_{z}$, but affects its amplitude and initial phase.

**Fig 9:**
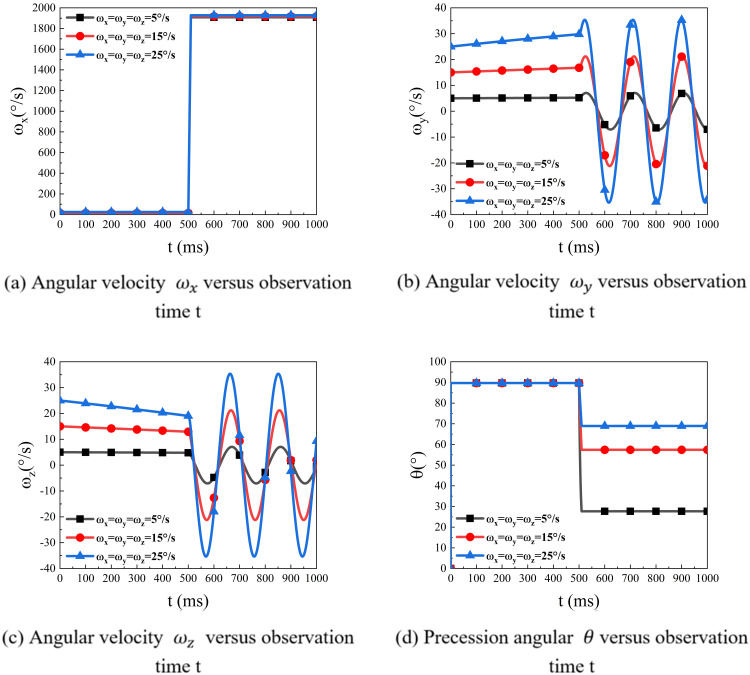
Influence of angular velocity of different values. **(a)** Angular velocity $\omega_{x}$ versus observation time t. **(b)** Angular velocity $\omega_{y}$ versus observation time t. **(c)** Angular velocity $\omega_{z}$ versus observation time t. **(d)** Precession angular θ versus observation time t.

In order to study the influence of the initial angular velocity on the micro-motion characteristics of the lightweight spacecraft, the angular velocities in each direction are set as 5°/s, 15°/s and 25°/s. Using the thrust of the second spinning rocket in Fig 4(a), the results shown in Fig 9 can be obtained.

As shown in Fig 9(b) and 9(c), before applying the spinning moment, the angular velocity in each direction influences each other and changes slightly in the angular velocity. With the operation of the spinning rocket, it enters the precession state. For angular velocities of different values, the amplitudes of $\omega_{y}\;{\text{and }\omega }_{z}$ are different, and the periods and initial phases are the same, and the corresponding precession angles also increase with the increase of the initial angular velocity.

**Fig 10:**
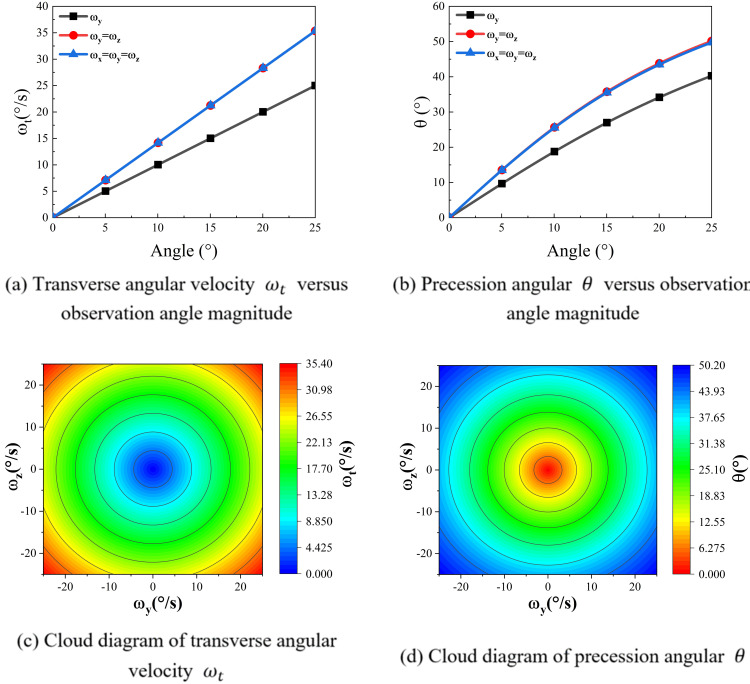
The influence of initial angular velocity. **(a)** Transverse angular velocity $\omega_{t}$ versus observation angle magnitude. **(b)** Precession angular θ versus observation angle magnitude. **(c)** Cloud diagram of transverse angular velocity $\omega_{t}$. **(d)** Cloud diagram of precession angular θ.

The above two analyses show in details the changes of the angular velocities of the main axes of inertia in the body coordinate system. In combination with equation (9), it can be found that when the spinning angular velocity remains unchanged, the transversal angular velocity is the key to the precession angle. Therefore, the transversal angular velocity and precession angle are carefully studied. To calculate the transversal angular velocity and precession angle under the initial angular velocities of different directions and different values, the results are shown in Fig 10.

As shown in Fig 10(a) and 10(b), the transversal angular velocity and precession angle also increase with the increase of angular velocity in all directions, and there is a linear relationship between the transversal angular velocity and initial angular velocity. The precession angle increases with the increase of initial angular velocity, but its increasing speed decreases gradually. The increase and decrease of the transversal angular velocity would obviously change the precession angle of the lightweight spacecraft, while the addition of the angular velocity $\omega_{x}$ would not have an obvious effect on the transversal angular velocity and precession angle of the lightweight spacecraft. As shown in Fig 10(c) and 10(d), the contours of the cloud map are circular. With the increase of the angular velocity in both directions, the contours of $\omega_{t}$ change uniformly, and the contours of θ gradually become sparse. The closer to the center of the circle the location is, the greater transversal angular velocity is, and the smaller the precession angle is. The further away from the center of the circle the location is, the greater the transversal angular velocity is, and the greater the precession angle is. An initial angular velocity deviation of 25°/s in one direction would produce a precession angle of 40.2° for the test device, and an initial angular velocity deviation of 25°/s in multiple directions would produce a precession angle of 45.7°. The main reason is that when $I_{t}\gg I_{x}$ in the test device and the real lightweight spacecraft, a small initial angular velocity deviation would produce a large precession angle, which seriously affects the spin stability of the lightweight spacecraft. Therefore, in the actual launching process, the transversal interference should be minimized.

## Conclusions

In this paper, in order to study the influence of different initial angular velocity deviations on the motion attitude of the lightweight spacecraft, a micro-motion characteristic experiment device is designed, and the micro-motion characteristic test is carried out. A dynamic model of the motion of the test device is established and the motion attitude of the test device is simulated. The accuracy of the model is verified by comparing the numerical simulation results with the experimental results. The simulation calculations of the motion attitude of the test device under different initial angular velocity deviations are carried out, and the following conclusions are drawn:

(1) The transversal angular velocity is the main factor affecting the precession angle of the lightweight spacecraft, and the spin angular velocity generated during launching has little effect on the precession angle of the lightweight spacecraft. Compared with one transversal angular velocity interference, the periods of two transversal angular velocities are unchanged, and the amplitude and initial phase change.
(2) When the direction of the initial angular velocity is determined, the transversal angular velocity is linear with the initial angular velocity, and the precession angle increases with the increase of the initial angular velocity. The precession angle increases with the increase of the deviation of the initial angular velocity, but its increasing speed would gradually decrease.
(3) The contour lines of the cloud image about the transversal angular velocity and precession angle are circular under two different directions of initial velocities. When the location is closer to the center of the circle, the values of the transversal angular velocity and precession angle become smaller.

## Supporting information

S1 Dataset S1_dataset.zip. (ZIP)

S1 File Simulation dataset. (XLSX)
